# Design and Performance Verification of Bionic Octopus Sucker Sealing Structure for Solenoid Valves

**DOI:** 10.3390/biomimetics10070425

**Published:** 2025-07-01

**Authors:** Zhihong Wang, Xinbin Zhang, Zhengzhi Mu, Xiang Guan, Junchi Liu, Zhipeng Pan, Junchong Wang, Xiangrui Ye, Zhenghai Qi, Jianyang Dong, Yongming Yao, Liucheng Zhou

**Affiliations:** 1 School of Mechanical and Aerospace Engineering, Jilin University, Changchun 130022, China; zhihong23@mails.jlu.edu.cn (Z.W.); guanxiang22@mails.jlu.edu.cn (X.G.); liujc22@mails.jlu.edu.cn (J.L.); panzp23@mails.jlu.edu.cn (Z.P.); junchong23@mails.jlu.edu.cn (J.W.); yexr24@mails.jlu.edu.cn (X.Y.); qizh24@mails.jlu.edu.cn (Z.Q.); jydong24@mails.jlu.edu.cn (J.D.); 2 National Key Laboratory of Aerospace Power System and Plasma Technology, Air Force Engineering University, Xi’an 710038, China; xinbin110@foxmail.com; 3 Key Laboratory of Bionic Engineering, Ministry of Education, Jilin University, Changchun 130022, China; zmu@jlu.edu.cn

**Keywords:** bionic sealing technology, octopus sucker morphology, annular groove structure, solenoid valve poppet, pull-out test

## Abstract

Aiming at the problem of the insufficient sealing performance of the solenoid valve poppet under a high working load and inspired by the multilevel groove structure of the octopus sucker and the adaptive sealing mechanism, a bionics-based design scheme for an annular groove sealing structure is proposed. By extracting the microscopic groove morphology features of the octopus sucker, we designed a multilayer rectangular cross-section groove structure at the annular interface, combined the designed structure with the Abaqus cohesive model to simulate the interface stripping behavior, and verified its mechanical properties by the pull-out test. The results show that the bionic groove structure significantly improves the bearing capacity of the sealing ring by enhancing the interface contact stress distribution and delaying the crack extension. Under the same working condition, the bionic structure increases the pull-out force by 46.1% compared with the traditional planar sealing ring. This study provides bionic theoretical support and an engineering practice reference for the design of sealing structures in complex working conditions, such as the solenoid valve poppet.

## 1. Instruction

Sealing technology [1,2] is crucial in the engineering field as it directly affects the operation, safety, and life of equipment [3,4,5,6].

Traditional static seals include gaskets, O-rings, and packing seals [7]. Gaskets rely on bolt tightening and deformation to fill a gap and are used for pipe connections. O-rings are used for pressure sealing and are suitable for both static and dynamic applications [8,9]. Packing seals are simple in structure and general in performance and are commonly used in small pumps [10,11,12]. Dynamic seals include mechanical seals, labyrinth seals, floating ring seals, etc. [13,14,15,16]. Mechanical seals rely on springs to make the sealing surface fit and are used in rotating equipment. Labyrinth seals use staggered teeth and gap throttling and are used in turbines [17,18,19,20]. Floating ring seals rely on oil film lubrication and are used in high-pressure centrifugal pumps [21,22].

In the field of aviation, the sealing reliability of the solenoid valve spool is directly related to flight safety, and traditional rubber sealing rings and metal gaskets have difficulty in meeting sealing needs under high-load and strong-vibration environments due to their fast aging, poor adaptability to complex working conditions, and other defects. Nature provides multiple inspirations for sealing technology innovation: geckos achieve efficient adsorption through the van der Waals force of their foot bristles [23,24,25], but this nanoscale mechanism of action is difficult to adapt to the roughness of a macro-engineering interface; the protein adhesive secreted by mussels can adhere to complex media [26] but is limited by the material’s durability; the fluid damping structure of the carp’s forked tail has inspired the design of bionic grooves in dry gas seals, but such designs are not as simple as the carp’s tail structure. The fluid damping structure of the carp forked tail inspired the design of bionic grooves in dry gas seals [27], but these are only applicable in single-phase airflow environments. The clustered helical grooves in the wings of a flying bird enhanced membrane stiffness at high speeds and low pressures [28,29] but failed to solve the problem of stress concentration in low-speed and high-pressure scenarios. The grooved biomimetic suction cups on the surface of the Kawarau Loach’s fins improved the adsorption force by 71.22% [30], and the prototype suction cups of the abalone’s ventral peduncle achieved a 15.8% enhancement in adsorption [31]. However, both relied on single-groove morphology optimization, failing to reveal the biological polymorphism or the synergistic mechanism of biological multistage sealing interfaces.

The central limitation of existing studies is the lack of cross-scale synergistic effects in the structure of biological multilevel grooves. Octopus suckers are extremely efficient adsorption and sealing organs in nature [32]. Their unique structure and extraordinary sealing ability are very prominent in the biological world. In-depth study of the octopus sucker structure and its application to bionic sealing design is of great significance for improving modern sealing technology performance.

In this paper, from a bionics perspective, the octopus sucker is studied for its excellent adsorption and adaptation to different morphological surfaces. The octopus sucker constructs multiple vacuum cavities with the help of micron-sized grooves and epidermal folds, thus realizing adaptable adsorption. Its groove distribution can effectively disperse stress and enhance interfacial friction. Based on this, this paper conducts adsorption simulation analysis and tensile experiments on a bionic structure. By deeply analyzing the experimental results and adsorption mechanism, this study aims to provide new insights for enhancing sealing efficiency and significantly improving sealing performance.

## 2. Morphological Feature Extraction and Bionic Design

### 2.1. Analysis of Octopus Sucker Morphology

Among the many biological objects that can be studied, the octopus is an ideal choice for in-depth investigation of the structure of the sucker because of its wide distribution and relatively large size, which makes it easy to observe and study.

In order to understand the microscopic and macroscopic features of the octopus’s sucker, two advanced observation tools, namely, a body microscope and a scanning electron microscope, were used to observe the sucker in great detail. With the deepening of observation, the unique and mysterious shape of the octopus sucker was gradually presented to the world.

**Figure 1:**
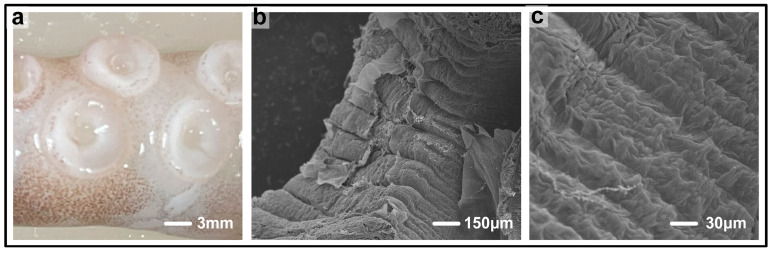
Octopus sucker structure observation. (**a**) Macroscopic view of octopus tentacle, showing single groove on sucker surface. (**b**) SEM (S-3400N) micro-view of sucker surface, with numerous grooves. (**c**) Another SEM micro-view, presenting more detailed groove features.

By observing the tentacle of an octopus, as shown in Figure 1a, we can clearly see that there is a single macroscopic groove structure on the surface of the sucker of the octopus tentacle. After further magnification with a scanning electron microscope (SEM, S-3400N, Hitachi High-Tech, Tokyo, Japan), we can see a large number of grooves on the surface of the sucker, as shown in Figure 1b,c. These grooves and protrusions are not randomly distributed but exhibit a herringbone or circular pattern. This unique groove structure is likely the key to the excellent adsorption performance of the octopus, and it may hide important mechanisms related to the adsorption process.

Upon examining the overall structure of the octopus sucker, we find that the construction of its adsorption space is exquisite. It is an organic whole composed of several different parts that cooperate with each other. Specifically, it includes the fibrous tissues at the top of the sucker, which may play a certain supportive and stabilizing role in the adsorption process; the upper cavity of the sucker, which provides the necessary spatial environment for the adsorption process; the funnel-shaped capacitive cavity, whose unique shape and design may contribute to the flow of liquids or gases, thereby affecting the adsorption force; the orifice, which is a key part of the contact with the object to be adsorbed; the annular muscular layer, which regulates the adsorption force through muscle contraction and diastole; and the rough fibrous tissue on the surface of the sucker and the wall fibrous tissue, which also play important roles in the adsorption process. Each of these parts has a unique function, and they cooperate with one another to realize the strong and stable adsorption capacity of the octopus sucker [33].

During in-depth study of octopus suckers, further observation reveals that the non-smooth grooves on the bottom surface of octopus suckers are rich and diverse in terms of morphology, mainly presenting in radial linear, herringbone-shaped, and transverse circular forms. These different groove structures may play unique roles in adsorbing different types of objects and adapting to different environmental conditions.

In order to quantitatively analyze the pit structures on the surface of octopus tentacle suckers more scientifically and accurately, a series of rigorous measurements were taken. First, for each scanning electron microscope (SEM) image, the number of pits in the area covered by two diagonal lines with a width of 5 μm was measured, and the diameter of the pits (expressed as the average of their long and short sides) was also measured. In this way, it was possible to obtain important information such as the distribution density and the size of the pits at the interface. Meanwhile, in order to accurately measure the depth and width of the pits on the surface of the suckers, a fully automated body-view microscope self-measurement system was used. Ten carefully selected octopus suckers were measured under the same field-of-view conditions to ensure the accuracy and comparability of the measurement results. After a series of rigorous measurement operations, the data presented in Table 1 were obtained.

Octopus suckers have a unique multistage sealing groove structure, and their adsorption capacity is extremely strong. On the surface of the octopus sucker, there are a large number of linear and circular grooves, and the bottom surface also has linear and circular, non-smooth groove forms. When the sucker is close to the surface of the adsorption object, the adsorption process will be divided into several layers to gradually realize efficient adsorption and sealing.

The outermost grooves are the first line of defense in the multistage sealing structure. At the moment of contact with an object, they take the lead in adhering to the surface of the object over a large area, quickly squeezing out the air or liquid in the grooves, forming a preliminary pressure difference, realizing the initial sealing, and laying the foundation for subsequent closer adsorption. As the suckers get closer to the surface of the object, a large number of inner grooves come into play. These inner grooves will closely fit the subtle undulations of the object’s surface, further extruding air or liquid, increasing the pressure difference, and significantly increasing the adsorption force. This is equivalent to the intermediate reinforcement layer in a multistage seal, which strengthens both the adsorption effect and the sealing effect. At the same time, the grooves also provide support and friction from multiple directions, preventing the sucker from sliding on the surface of the object and stabilizing the adsorption state.

Inspired by the structure of the octopus sucker, we optimized the design of the sealing groove. In terms of shape, linear and circular grooves were designed to mimic an octopus sucker. The circular grooves are similar to the lateral circular grooves of the octopus sucker, which can form multiple annular pressure zones on the sealing surface, like a sealing barrier, preventing the leakage of medium in all directions and enhancing the adhesion stability of the seal. The linear groove guides the flow of sealing medium, forming a pressure difference on both sides, increasing the friction between the seal and the sealed object, and helping to stop the leakage of the medium.

Different shapes and sizes of grooves can realize different degrees of sealing and anti-peeling effects when the seals are working. The grooves mimic the action of the microstructure of an octopus sucker, providing a fine seal, blocking tiny medium leaks, and generating stronger pressure differences and friction to cope with more demanding sealing environments. The arrangement of the grooves also draws on the characteristics of the octopus sucker. Setting the spacing and number of grooves in a reasonable manner ensures an even distribution of pressure on the sealing surface. When the seal is subjected to external forces trying to peel it, multiple grooves work together, just like an octopus sucker, to resist the peeling of external forces, effectively dispersing the peeling force, significantly improving the seal’s anti-peeling ability, and ensuring reliable sealing performance. In order to further optimize the design, the adsorption capacity of different sizes of linear and circular grooves can also be compared so as to select the appropriate shape, size, and distribution of grooves.

### 2.2. Parameterized Design of Bionic Structures

For solenoid valve poppets with high-precision micro-components, the structure of the octopus sucker cannot be directly applied with its original dimensions. Mature octopus sucker groove widths and depths are in the range of 0.1–0.5 mm. If the surface grooves of the metal pressure-sealing groove are bionically designed according to this standard, the roughness of the model surface will be too great, which will not only destroy the structural strength of the component but also not be conducive to the study of the role of non-smooth grooves in the sealing performance.

Therefore, according to the basic parameters of the pneumatic solenoid valve, based on the concept of shape bionics, we reasonably scaled the non-smooth morphology parameters of the octopus sucker. After research, the groove widths and depths were set to three categories: 0.02 mm, 0.04 mm, and 0.08 mm. This not only endowed the bionic sucker with an obvious non-smooth morphology but also did not seriously affect the structural strength of the valve.

Regarding the recess layout design, due to the difference in the groove deformation ability between the metal valve pressure-glue groove and the octopus sucker, we could not copy the groove density of the octopus sucker. Changing the number of grooves around the bottom surface of the sucker can not only adjust the density of the non-smooth form but also facilitate mold processing. So, the number of grooves on the inner surface of the sucker can be used as a reference to measure the degree of non-smoothness.

**Figure 2:**
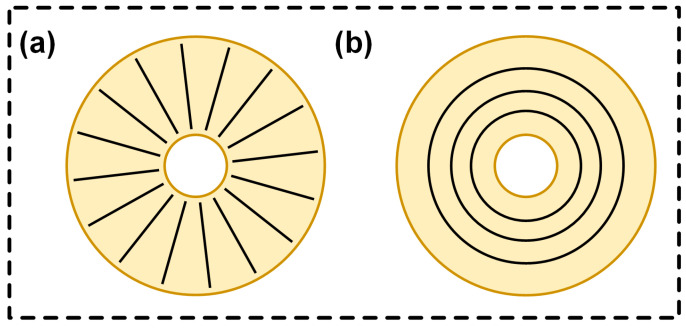
Schematic diagram of (**a**) the linear grooves and (**b**) the circular grooves.

According to the basic size of the metal valve pressure-glue groove, in addition to the characteristic that the number of more obvious grooves on the surface of a mature octopus sucker is 16–21, the design of the number of grooves followed the principle of the reasonable distribution of the number of non-smooth grooves, with each set having three levels. The number of circumferential linear grooves arranged around the axis was 16, 32, and 48 (schematic diagram in Figure 2a); circular grooves were radially distributed on the inner surface of the glue groove (schematic diagram in Figure 2b), and the number of circular grooves was set to 4, 6, and 8. Each type of groove was uniformly distributed at equal intervals on the inner surface of the glue groove.

Based on the above design ideas, the design specifications of the bionic recess for the metal-flap pressure-glue groove are listed as follows in Table 2.

### 2.3. Bionic Model Construction

**Figure 3:**
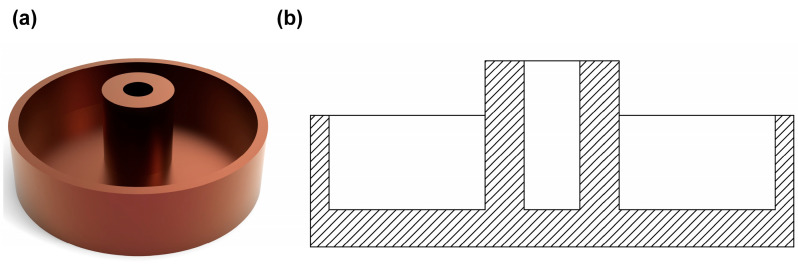
Sealed valve plate. (**a**) Sealed valve model drawing. (**b**) Sealed valve section.

In the exploration of valve seal simulation design, the study focused on the valve prototype. Considering the practical application scenarios and the feasibility of simulation, the valve prototype was moderately simplified. During the simplification process, the original key parameters were strictly retained to ensure the accuracy and credibility of the simulation results. In order to optimize the sealing performance of the trapdoor, the research cleverly drew on the concept of bionics and started to build a characteristic bionic structure on the surface of the trapdoor. With the help of precision machining, micron-sized grooves were added on the surface of the flap to simulate the fine texture of the octopus sucker surface, which not only enhanced the surface friction but also helped to create a local vacuum environment. At the same time, referring to the octopus epidermal fold pattern, corresponding fold structures were sculpted on the surface of the valve, which, together with the micron-sized grooves, successfully formed multiple vacuum cavities. After completing the structural design, the model was further simplified according to the precise structural parameters, and then professional 3D modeling software was used to successfully build a 3D model of the valve, the detailed structure of which can be seen in Figure 3a,b, which served as a solid foundation for the subsequent in-depth simulation and analysis of the valve seal.

## 3. Results and Analysis of Finite Element Simulation and Experiments

### 3.1. Abaqus Model Construction

The material properties were modeled using the rubber hyperelastic (Mooney–Rivlin) model, and cohesive units were embedded within the interfacial layer to simulate the peeling process. In the finite element calculation, the damage of the cohesive model was based on the stress–displacement separation criterion. According to this criterion, it is assumed that the material is a linear elastic material. The material units can be separated through tensile separation along the thickness direction, shear separation perpendicular to the thickness direction, or a mixed mode of these two types of separation.

**Figure 4:**
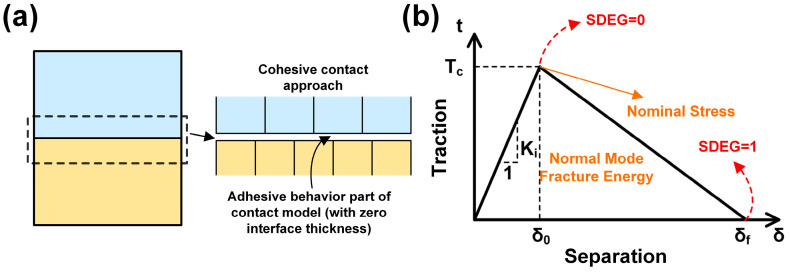
(**a**) Cohesive contact approach. (**b**) Diagram of the triangular separation criterion.

Four cohesive damage criteria exist: the maximum nominal stress criterion, the maximum nominal strain criterion, the secondary nominal stress criterion, and the secondary nominal strain criterion. In this paper, the maximum nominal stress criterion was adopted for simulation. The maximum nominal stress criterion has three damage modes: triangular, exponential, and trapezoidal. The following is an example of the triangular traction–separation criterion. Figure 4a shows the cohesive contact approach, and (b) is a diagram of the triangular separation criterion.

The key assumptions of the cohesive model require further clarification:

(1) The introduction of a zero-thickness adhesive layer was intended to simulate the “ideal contact” characteristics of the sealing interface, avoiding errors caused by artificially setting thickness during geometric modeling. This approach aligned with the actual interface contact state during the assembly of the sealing ring.
(2) The maximum nominal stress criterion was chosen because interface failure in pull-out tests was observed to initiate in regions of normal stress concentration. This criterion uses normal stress as the damage initiation index, which is highly consistent with experimental observations.
(3) The linear softening characteristics of the triangular traction–separation criterion (as shown in Figure 4b) can succinctly describe the progressive process from elastic deformation to complete failure of the interface. Compared to exponential or trapezoidal criteria, the triangular model allows parameters to be directly fitted using the stress–strain curve from tensile tests, requiring fewer computational iterations and achieving better convergence efficiency.

In the material properties module of the finite element software ABAQUS 2018, E refers to the elastic modulus of the cohesive element, $K_{i}$ refers to the stiffness of the cohesive element, $\delta_{0}$ is the displacement corresponding to the onset of damage of the cohesive element, $\delta_{f}$ is the displacement corresponding to the complete failure of the cohesive element, $T_{c}$ is the maximum tensile stress corresponding to the cohesive element, and $\varepsilon_{f}$ is the strain corresponding to the complete failure of the cohesive element. $\varepsilon_{0}$ is the strain corresponding to the damage initiation of the cohesive element, and $h_{e}$ is the thickness of the cohesive unit.

To illustrate the tensile separation criterion along the thickness direction, the stiffness of the cohesive unit is expressed as follows:

(1) $$K_{i}=T_{c}{/\delta }_{0}$$

where the tensile stress, N, is defined by the following equation:

(2) $$N=E_{n}\varepsilon_{n}$$

where the tensile strain is defined as $\varepsilon_{n}=\delta_{n}/h_{e}$.

Then it is known that

(3) $$E_{n}=K_{n}h_{e}$$

From the above equation, it can be seen that there is a relationship between the elastic modulus and the stiffness of the cohesive element, and the thickness of the cohesive unit will affect the stiffness of the unit.

In a specific simulation, the adhesive layer is set as a cohesive element with zero thickness, and the bonding unit has only one layer along the thickness direction. SDEG represents the damage factor of the unit, i.e., when the damage factor reaches a certain value, the unit will fail. The minimum value of the damage factor is 0, i.e., no damage, and the maximum value is 1, i.e., complete damage failure. When the damage factor reaches 1, this means that the strain energy release rate of the unit is equal to the cracking energy of the unit, and the value of the cracking energy is equal to the area enclosed by the stress–displacement curve and the x-axis. Based on the feasibility of the calculation, the maximum value of the damage factor is set to 0.99.

The elastic modulus of the rubber material is 7.84 MPa, and the Poisson’s ratio is 0.47. The elastic modulus of the bronze material is 110,000 MPa, and the Poisson’s ratio is 0.33. The elastic parameters of the cohesive unit are as follows: E/Enn=6.809 MPa, which represents the elastic modulus in the normal direction of the unit; G1/Enn=6.809 MPa; and G2/Ett=6.809 MPa. The maximum damage parameter (nominal stress) is 0.35, the viscosity coefficient is 0.0001, and the fracture energy is 0.09.

### 3.2. Experimental Setup and Procedures

#### 3.2.1. Testing Platform

Mechanical test equipment: A high-precision electronic universal testing machine was chosen. This equipment has a stable loading system and an accurate stress measurement function. Its measurement accuracy can reach ±0.5%, such that it can meet the loading requirements of different structural samples in the pulling test and accurately record the stress changes during the pulling process. It is equipped with a displacement sensor with a resolution of 0.001 mm, which can monitor the displacement of the sample in real time during the drawing process for analyzing the stress–strain relationship.

Sample fixtures: Customized fixtures were designed for sealing structure samples of different shapes and sizes, such as non-grooved interfaces, ray-grooved structure interfaces, circular-grooved structure interfaces, etc. These fixtures could firmly install the samples during the testing process and ensure uniform force application, thus avoiding deviations in test results caused by installation problems. The fixtures were made of high-strength aluminum alloy through precision machining and surface treatment to ensure their dimensional accuracy and stability.

Environment simulation system: Considering the complexity of the actual working environment of the solenoid valve poppet, the environment simulation box is capable of simulating typical working conditions (e.g., 80 °C, 60% RH) for aviation solenoid valves. The temperature control range was −50 °C to 150 °C, and humidity ranged from 20% to 95% RH, with extended tests for extreme conditions planned as future work. Through real-time monitoring by temperature and humidity sensors and feedback control, the environment simulation box could simulate the environment of the valve of a fighter aircraft in different flight conditions and test the performance changes of the sealing structure under the influence of different environmental factors.

#### 3.2.2. Test Process

Sample preparation: According to the bionic structure design program, the sealing structure samples with different structures and parameters were made by a precision machining process, including non-grooved interfaces, ray-grooved structure interfaces, circular-grooved structure interfaces, etc. The samples were made of rubber and bronze, which are similar to the actual materials used in the solenoid valve poppet, ensuring the reliability of the experimental results. Each sample part was numbered and dimensioned, and the initial data were recorded.

Installation of sample parts: The manufactured sample parts were installed on the customized fixture to ensure that the sample parts were installed in the accurate position and firmly fixed. For samples with different shapes, such as circular-grooved structure samples, we made sure that their center was aligned with the center of the fixture to avoid eccentric force. During the installation process, we checked the contact between the sample and the fixture to ensure that there was no gap or looseness.

Setting test parameters: The parameters of the pull-out test were set according to the purpose of the experiment and the characteristics of the sample on the electronic universal testing machine. The loading rate was set at 1 mm/min to ensure that accurate mechanical property data were obtained under quasi-static conditions, and the upper limit of displacement was set to prevent the sample from overstretching, which could have led to damage and affected the test results. At the same time, the environmental parameters were set to be representative of aviation applications (80 °C, 60% RH) in the simulation box, as these conditions reflect common operational loads on solenoid valve poppets.

Conduction of the pull-out test: The electronic universal testing machine was started and the pull-out force was applied to the sample piece according to the set loading rate. During the test, stress and displacement data were collected in real time by the force transducer and displacement transducer, and the data collection frequency was set to 10 Hz to ensure that a detailed stress–strain curve could be obtained. We closely observed the deformation of the sample. The stress and displacement data were recorded when the sample showed obvious deformation, cracking, or failure.

Data recording and analysis: After the test, we organized the collected data and drew the stress–strain curves for the samples of different structures. We analyzed the characteristics of the curves and calculated the mechanical property indexes of the samples, such as the tensile strength and the modulus of elasticity. We compared the test data for different structural samples to assess the differences in sealing performance and mechanical properties under different environmental conditions and provided data support for subsequent structural optimization.

Repeated tests: To ensure reliability and accuracy, four independent specimens were tested for each interface type (smooth, sandblasted, ray-grooved, and circular-grooved), exceeding the minimum requirement of three tests to validate statistical significance. Between each test, we checked the condition of the sample and the fixture, replacing them in time if they were damaged or abnormal. Statistical analysis of the data from multiple tests was performed to calculate the means and standard deviations to ensure the stability and credibility of the data.

### 3.3. Simulation Results and Experimental Analysis

#### 3.3.1. Simulation Results

**Figure 5:**
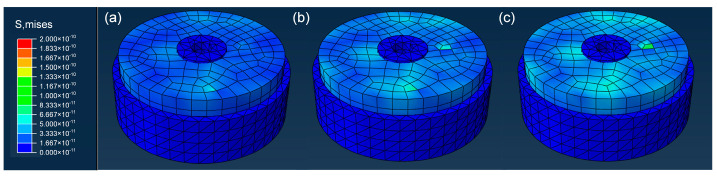
Three-dimensional finite element cohesive interface simulation. (**a**) [stage 1], (**b**) [stage 2], and (**c**) [stage 3].

In order to comprehensively analyze the mechanical properties of different planar structures, the research team carried out in-depth comparative finite element structural analyses and calculations for the non-grooved planar surface, the ray-grooved structural surface, and the circular-grooved structural surface. The whole process strictly followed the industry standards and norms, using advanced finite element analysis software to accurately construct the digital models of the three structures and to set boundary conditions and loading parameters that were highly suitable for the actual working conditions to ensure that the simulation results were real and reliable. After performing calculations, the simulation effects were visualized, as presented in Figure 5, which shows the stress distribution at three stages of the simulation: (a) [stage 1], (b) [stage 2], and (c) [stage 3], where the stress distribution is clearly identifiable. Moreover, the specific stress results were sorted in Table 3, providing key data support for the subsequent in-depth discussion and facilitating a precise understanding of the characteristics, advantages, and disadvantages of each structure in terms of stress bearing.

**Figure 6:**
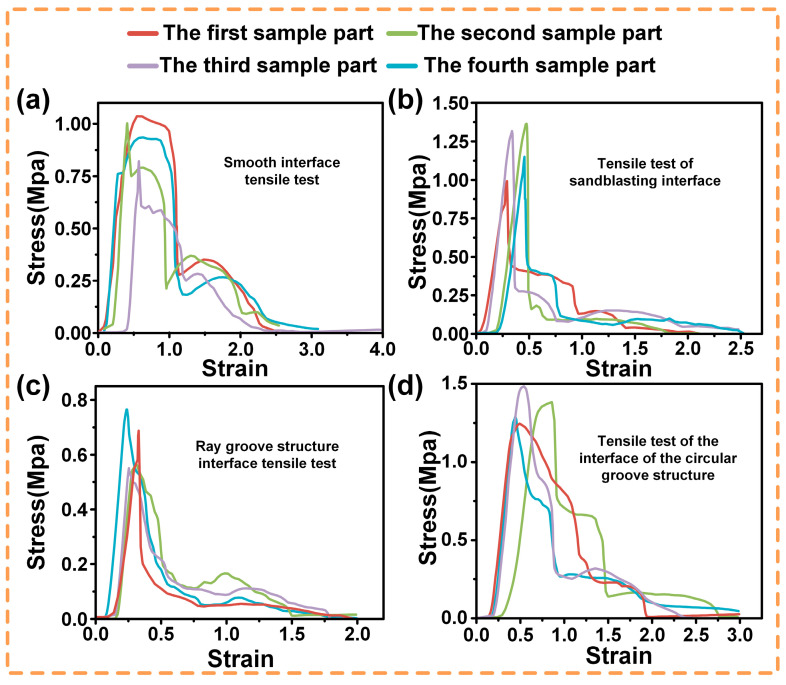
Stress–strain curves of four independent specimens per interface type: (**a**) smooth interface; (**b**) sandblasted interface; (**c**) ray-grooved interface; (**d**) circular-grooved interface.

Simulation results indicate that the circular-grooved structure interface excels in key performance metrics—maximum contact stress, stress distribution uniformity, and peeling resistance—demonstrating remarkable application potential. Specifically, its maximum contact stress reaches 1.217 × 10^3^ MPa (Table 3), 4.5-fold higher than the smooth interface (2.695 × 10^2^ MPa), while forming annular pressure zones (Figure 5) that evenly disperse stress to delay crack propagation. This structural design enhances peeling resistance by 46.1% compared to traditional planar seals, as validated by pull-out tests (detailed in Section 3.3.2). Given these superior mechanical and sealing properties—including uniform stress distribution and enhanced interfacial interlocking—the research team proceeded to validate the structure via actual machining tests under realistic conditions. After the precision machining was completed, the molded products were subjected to a pull-out test. Through the rigorous test process and data collection, the research team obtained comprehensive and detailed performance evaluation data, as shown in Figure 6.

#### 3.3.2. Experimental Results

To systematically study the tensile behaviors, uniaxial tensile tests were performed on four specimens for each of four interface types: smooth, sandblasted, ray-grooved, and circular-grooved. The tests adhered to standards, with strict control over conditions and parameters for data accuracy.

As shown in Figure 6:

In sub-plot (a) for the smooth interface, four specimen parts differ. The red curve rises fast in the elastic stage, peaking at about 1.00 MPa but entering the plastic stage early. Other curves also show varied elastic–plastic behaviors, likely due to manufacturing or material issues.

Sub-plot (b) of the sandblasted interface shows higher overall stress. The red curve rises sharply at first, peaking near 1.20 MPa. Sandblasting-induced roughness boosts bonding, and specimens resist deformation well, with minor differences among parts in plastic-stage details.

For the ray-grooved interface in sub-plot (c), the structure acts as a stress concentrator early. The red curve peaks around 0.7 MPa, then drops fast in plastic deformation. Groove distribution causes different responses among specimens.

In sub-plot (d) of the circular-grooved interface, curves balance elastic and plastic behaviors. The red curve peaks at around 1.41 MPa. Its design distributes stress evenly, with specimen parts having similar trends but different key-point values.

**Figure 7:**
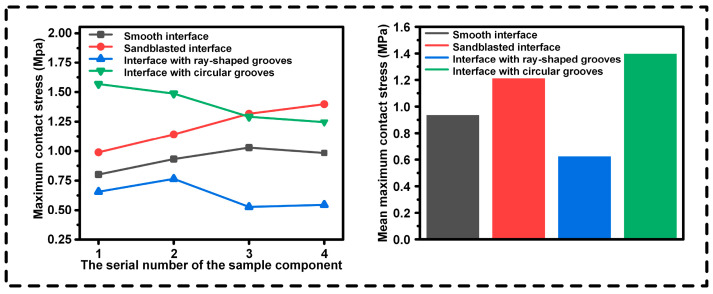
Analysis of the maximum contact stress of different contact interfaces.

We conducted an in-depth analysis of the tensile test results. In Figure 7, the maximum contact stresses at different interfaces of the four groups of specimens and their average maximum contact stresses are presented. The interface with circular grooves exhibits excellent bonding strength, reaching the level of the traditional sandblasted interface, which demonstrates its effectiveness in enhancing the stability of material connections. In contrast, the bonding strength of the ray structure is relatively poor, and there are obvious deficiencies in resisting tensile external forces.

Based on this conclusion, in order to further explore the performance potential of the ring structure, it was decided to embark on a new exploratory journey to carry out all-round modeling analysis and simulation optimization of the dimensions of the ring structure. With the help of advanced modeling software and sophisticated simulation algorithms, we meticulously studied how changes in the key dimensional parameters of the ring structure affected its performance and were committed to achieving a qualitative leap in the performance of the ring structure through precise dimensional control to provide a more optimized and efficient solution for real-world application scenarios.

### 3.4. Further Optimization: Investigating Different Cross-Sectional Shapes, Groove Depths, Groove Widths, and Quantities

Based on the optimal performance of the circular groove in Table 3, this section of the study optimized the cross-sectional shapes of the grooves (rectangular/triangular/arc-shaped). The baseline case without grooves was consistent with the “smooth interface” in Table 3 to ensure data comparability.

**Figure 8:**
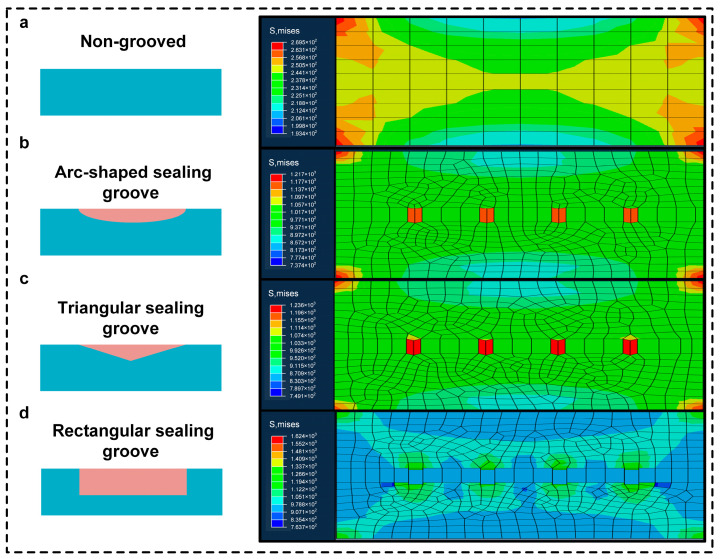
The results of the simulation analysis of (**a**) the non-grooved interface, (**b**) the curved-groove interface, (**c**) the triangular-grooved interface, and (**d**) the rectangular-grooved interface.

To investigate the mechanical response, load-carrying capacity, and stability of circular structures under different working conditions, we comprehensively and systematically analyzed circular structures with different cross-sectional shapes. The purpose was to reveal the differences in internal stress distribution and the failure modes of circular structures with different cross-sectional shapes during pull-out simulation, thereby providing a solid theoretical basis and data support for optimizing circular structure design and improving their comprehensive performance. The results of the simulation analysis of the non-grooved interface, curved-grooved interface, triangular-grooved interface, and rectangular-grooved interface are shown in Figure 8 and Table 4.

Stress Distribution and Failure Mode Analysis:

Non-grooved: Uniform stress (2.695 × 10^2^ MPa); fails by early interfacial peeling due to lack of geometric interlock.

Arc-shaped: Stress concentrates on curved edges (1.217 × 10^3^ MPa); gradual peeling along contours.

Triangular: Sharp tips induce stress peaks (1.236 × 10^3^ MPa); cracks initiate at corners.

Rectangular: Highest stress (1.624 × 10^3^ MPa) with uniform distribution on vertical walls; fails via cohesive failure in substrate/adhesive, not the interface.

Key mechanism: The rectangular grooves’ 4:1 depth–width ratio maximizes the side surface area, enhancing mechanical interlocking, like octopus sucker microstructures. This shifts failure from interfacial debonding to bulk material failure, boosting the pull-out force by 46.1%.

Following stress–failure mode analysis, the rectangular cross-section was chosen for its optimal load dispersion and cohesive failure behavior, aligning with high-load sealing requirements. The rectangular cross-section can effectively guarantee the good sealing of the sealing medium and minimize the risk of leakage when dealing with specific working conditions; when subjected to external pressure, its internal stress distribution is more reasonable, which can significantly improve the structural stability and durability of the sealing groove; at the same time, from the perspective of machining and manufacturing, the rectangular cross-section has a relatively mature and simple machining process, which can help to reduce the cost of production and improve the production efficiency, so it is the best choice for the current design.

**Figure 9:**
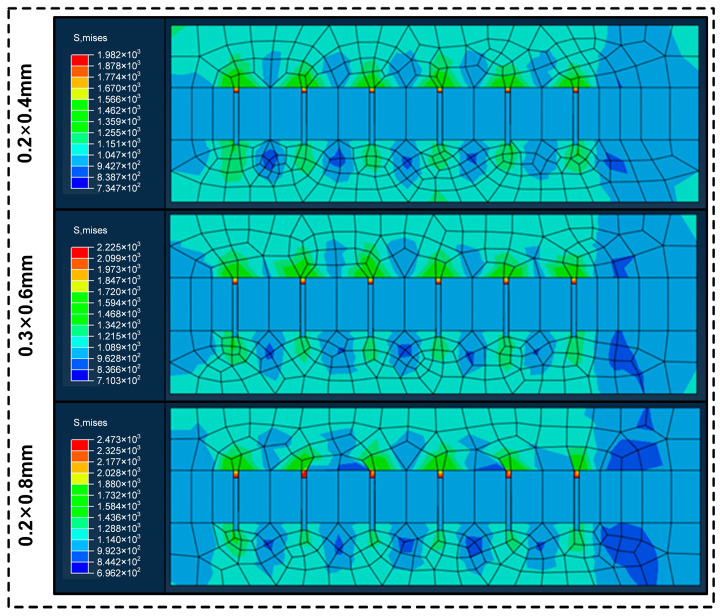
Simulation results for the rectangular sealing groove with dimensions of 0.2 mm × 0.4 mm, 0.3 mm × 0.6 mm, and 0.2 mm × 0.8 mm.

After selecting the rectangle as the cross-sectional shape for the sealing groove, in order to further improve the sealing performance, three key parameters, namely, the depth of the groove, the width of the groove, and the number of grooves, needed to be analyzed in depth, and a full factorial test was carried out with a total of 27 combinations to determine the optimal combination. In the research process, simulation software was used to simulate and calculate the different parameter combinations, taking into account the sealing effect, structural stress distribution, overall stability, and other indicators. After several rounds of simulation and comparison, three groups of parameters (groove depth × groove width: 0.2 mm × 0.8 mm, 0.3 mm × 0.6 mm, and 0.2 mm × 0.4 mm) with excellent effects were finally selected. These dimensions were chosen to represent distinct depth–width ratios (0.25, 0.5, and 1.0) for visualizing stress–strain behaviors across geometric proportions. The optimal 0.08 mm × 0.02 mm parameters (4:1 ratio) were derived by extrapolating these trends to balance mechanical interlocking efficiency with the micro-manufacturing constraints of solenoid valve poppets. The corresponding simulation results for slot depth, slot width, and number of slots are shown in Figure 9. These simulation images intuitively reflect the excellent performance of the selected parameters under actual working conditions, providing reliable data support for the accurate design and manufacture of subsequent sealing grooves.

**Figure 10:**
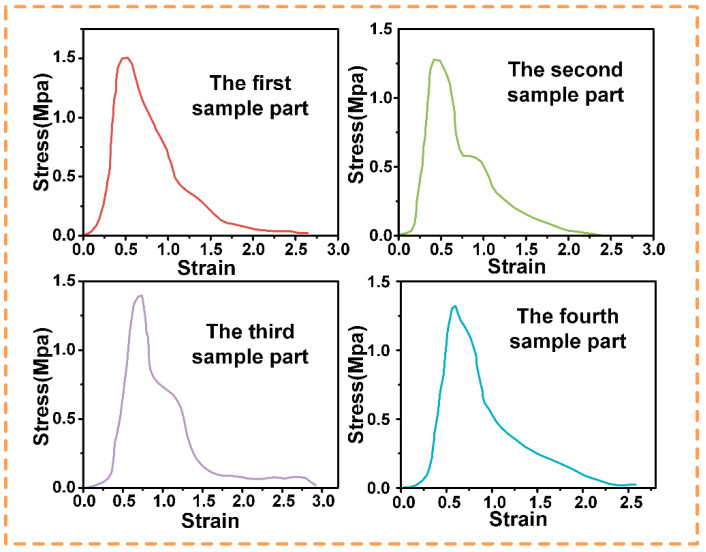
Stress–strain curve of the rectangular sealing groove with dimensions 0.2 mm × 0.4 mm.

**Figure 11:**
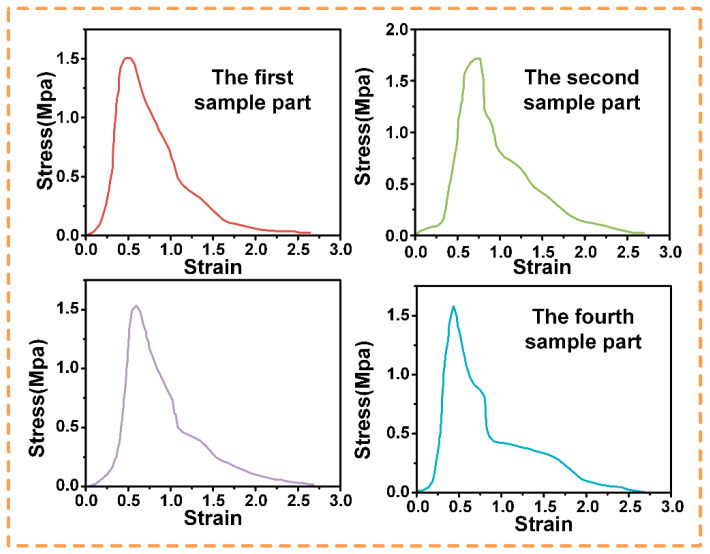
Stress–strain curve of the rectangular sealing groove with dimensions 0.3 mm × 0.6 mm.

**Figure 12:**
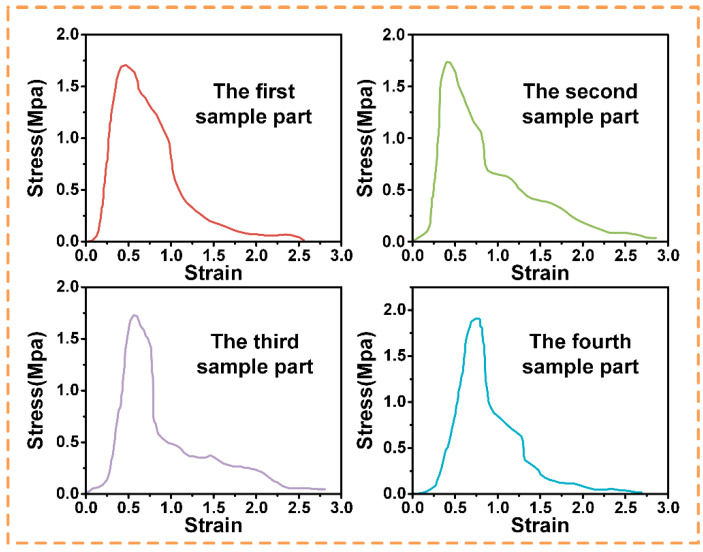
Stress–strain curve of the rectangular sealing groove with dimensions 0.2 mm × 0.8 mm.

In the subsequent study, actual pull-out tests were conducted to verify the effectiveness of the three sets of parameters selected, as shown in Figure 10, Figure 11 and Figure 12, and to determine the optimum parameters. In the adhesive pull-out test, the combination of a 0.08 mm groove depth, a 0.02 mm groove width, and six grooves performed the best. The slot depth of 0.08 mm, the maximum value within the test range, significantly increased the lateral surface area of the slot, allowing the adhesive to form a longer “anchored” structure which required greater shear forces to destroy it; at the same time, the depth was within the process control range, which reasonably balanced the structural strength.

Moreover, when the slot width is 0.02 mm, the capillary action of the adhesive can be utilized to completely fill the slot, reducing bubbles and voids and enhancing bond integrity; and with this narrow slot width, the proportion of the side area to the total surface area is greatly increased. For example, when the slot depth is 0.08 mm and the width is 0.02 mm, the proportion of the side area reaches 89%, which strengthens the overall performance of the embedment because the contribution of the side surface to mechanical embedment is much greater than that of the bottom surface. The 4:1 depth–width ratio (0.08 mm × 0.02 mm) was optimized from the stress distribution trends observed in Figure 9, where higher ratios consistently exhibited more uniform stress dispersion. This refinement differs from the representative ratios plotted in Figure 10, Figure 11 and Figure 12, as it integrates the mechanical “micro-mortise” interlocking effect with practical machining limits.

In terms of the number of slots, with the radial width of the ring of 2.1 mm, the distribution of six slots is the most reasonable. It not only overcomes the problem of insufficient surface area caused by having four slots but also avoids the situation where the spacing of eight slots is too small. (Too small a slot spacing may be regarded as a machining error and can reduce the strength of the substrate.) A moderate number of slots enables the load to be distributed uniformly, reduces the concentration of local stresses, and slows down the interfacial peeling.

In addition, a 0.08 mm depth and a 0.02 mm width form a 4:1 high depth-to-width ratio structure, similar to the “micro-mortise and tenon” structure, greatly enhancing the mechanical occlusion effect; with this parameter combination, the failure tends to occur inside the adhesive or substrate materials, rather than at the interface, indicating that the interface bond strength exceeds that of the adhesive layer or the substrate itself. This parameter combination shows excellent performance. By maximizing the side surface area, optimizing the quality of the adhesive filling, and reasonably distributing the stress, this parameter combination realizes the optimal balance between mechanical locking and interfacial strength, which leads to the best performance in the pull-out test.

**Figure 13:**
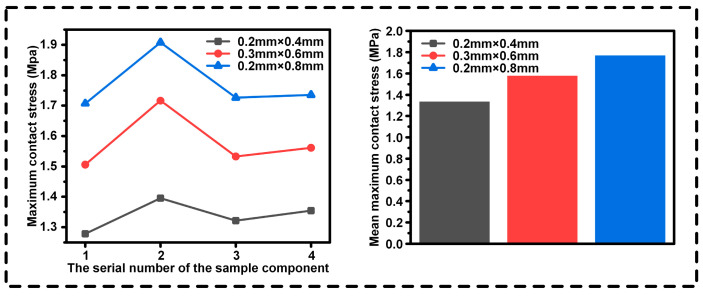
Analysis of the maximum contact stresses for different groove sizes.

We conducted an in-depth analysis of the results of the tensile test. In Figure 13, the maximum contact stresses at the interfaces with different groove parameters of the four groups of specimens and their average maximum contact stresses are presented. The condition of a groove depth of 0.08 mm and a groove width of 0.02 mm has the maximum contact stress and shows the best effect.

The final selected structure was a circular structure with rectangular grooves. It had a groove depth of 0.08 mm, a groove width of 0.02 mm, and a total of six grooves. Compared with the traditional sandblasting method, this structure can increase the surface stress strength by approximately 46.1%. Moreover, it provides a clear direction for optimizing the sealing structure of the fighter aircraft valve.

This structure achieves a good balance between mechanical locking, adhesive filling, and stress distribution, effectively improving the sealing performance and meeting the sealing requirements of the solenoid valve poppet under complex working conditions.

Based on this structure, subsequent research can conduct in-depth performance investigations under multi-physical field coupling by integrating the vibration and temperature alternation factors in the actual application scenario. Additionally, it can explore the compatibility of new materials with this structure, thereby continuously optimizing the sealing performance, offering stronger support for the development of aerospace sealing technology, and driving technological progress in related fields.

## 4. Conclusions

Based on octopus sucker observation tests, an annular planar sealing structure with multilayer rectangular cross-sectional grooves was designed, inspired by the octopus sucker’s multistage groove structure and sealing mechanism. The design parameters, such as the groove cross-sectional shape, width, length, and number, were studied. Abaqus and the cohesive model were used for simulation, and pull-out tests verified the results.

Rectangular cross-section grooves were found to be optimal. After experimental optimization, a groove depth of 0.08 mm, a width of 0.02 mm, and a total of six groves showed the best performance in pull-out tests. This combination balanced structure, adhesion, and stress, optimizing interfacial strength.

The bionic structure’s pull-out force was 46.1% higher than that of the traditional planar sealing ring. By mimicking the octopus sucker’s stress dispersion and friction enhancement, the bionic sucker’s adsorption and the sealing structure’s load-carrying capacity were improved. This bionic design offers a new concept for the engineering field.

Future work will expand to multi-physical field optimization, including validation across the full temperature range (−50 °C to 150 °C) and fluid-medium interactions, building upon the baseline performance verified at 80 °C/60% RH.

Concurrently, engineering applications will expand into aerospace (e.g., high-pressure/high-temperature/vibration-resistant seals for solenoid valves and rocket fuel systems), deep-sea equipment (enhancing high-pressure reliability for submersible robotic joints and pipeline connections), and medical devices (developing bioinspired groove-adaptive seals to improve interfacial adhesion and reduce infection risks in surgical applications).

## Figures and Tables

**Table 1:** Octopus sucker groove size table (mm).

Serial Number of the Octopus Sucker	Groove Depth, H (mm)	Groove Width, L (mm)
1	0.13	0.19
2	0.25	0.17
3	0.35	0.42
4	0.27	0.20
5	0.16	0.26
6	0.45	0.36
7	0.29	0.17
8	0.39	0.32
9	0.31	0.20
10	0.41	0.35

**Table 2:** Design scheme of bionic grooves for the glue groove of the metal valve.

Serial Number	Dimensions (Width, Depth (mm))	Shape	Number
1	0.02	Linear shape	16
2	0.02		32
3	0.02		48
4	0.04		16
5	0.04		32
6	0.04		48
7	0.08		16
8	0.08		32
9	0.08		48
10	0.02	Circular shape	4
11	0.02		6
12	0.02		8
13	0.04		4
14	0.04		6
15	0.04		8
16	0.08		4
17	0.08		6
18	0.08		8

**Table 3:** Simulation results for different groove morphologies (ray-grooved vs. circular-grooved) and non-grooved interface.

Type	Maximum Contact Stress (Mpa)
Smooth interface	2.695 × 10^2^
Interface of the ray-grooved structure	1.236 × 10^3^
Interface of the circular-grooved structure	1.217 × 10^3^

**Table 4:** Simulation results for different cross-sectional groove shapes (rectangular/triangular/arc-shaped) vs. non-grooved interface.

Type	Maximum Contact Stress (Mpa)
Non-grooved	2.695 × 10^2^
Rectangular sealing groove	1.624 × 10^3^
Triangular sealing groove	1.236 × 10^3^
Arc-shaped sealing groove	1.217 × 10^3^

## Data Availability

The original contributions presented in this study are included in the article. Further inquiries can be directed to the corresponding author.
